# The Trenbolo(g)ne Sandwich: An International Study Comparing Health Harms Among Men Who Use Anabolic‐Androgenic Steroids With and Without Trenbolone

**DOI:** 10.1111/dar.70162

**Published:** 2026-04-27

**Authors:** Benjamin Bonenti, Bahareh Ahmadinejad, Cheneal Puljević, Jason Ferris, Adam Winstock, Kyle T. Ganson, Jason M. Nagata, Adam Bradshaw, Timothy Piatkowski

**Affiliations:** ^1^ School of Public Health, Faculty of Health, Medicine and Behavioural Sciences The University of Queensland Brisbane Australia; ^2^ School of Applied Psychology and Griffith Centre for Mental Health Griffith University Brisbane Australia; ^3^ University College London London UK; ^4^ Global Drug Survey London UK; ^5^ Factor‐Inwentash Faculty of Social Work University of Toronto Toronto Canada; ^6^ Division of Adolescent and Young Adult Medicine, Department of Pediatrics University of California San Francisco San Francisco California USA; ^7^ Queensland Injectors Voice for Advocacy and Action Sunshine Coast Australia

**Keywords:** anabolic‐androgenic steroids, cardiometabolic harms, global drug survey, harm reduction, psychosocial harms, side effects, trenbolone

## Abstract

**Introduction:**

Trenbolone is a high‐risk anabolic‐androgenic steroid (AAS), yet quantitative evidence describing its psychosocial and physical harm profile remains limited. This study compared self‐reported concerns among men who used trenbolone in the past 12 months (trenbolone group) with those who used other AAS but not trenbolone (non‐trenbolone group).

**Methods:**

Data were drawn from male respondents to the Global Drug Survey 2024 who reported past‐year AAS consumption (*N* = 1146; *M*_age_ = 31.46, *SD* = 9.93). Participants were categorised as the trenbolone group (*n* = 237) or non‐trenbolone group (*n* = 909) based on past‐12‐month injectable trenbolone use. Chi‐squared tests examined between‐group differences in psychosocial and physical concerns. Multiple‐response frequencies and UpSet plots were used to assess number and co‐occurrence of reported harms.

**Results:**

Chi‐square analyses indicated that psychosocial concerns, including mood instability, irritability and depressive symptoms, were significantly more common among the trenbolone group (all *p* < 0.001), with small‐to‐moderate effect sizes (*ϕ* = 0.13 to 0.20). Physical concerns, particularly cardiovascular and hepatic effects, were also significantly more prevalent among the trenbolone group (all *p* < 0.001; *ϕ* = 0.18 to 0.20). UpSet plot visualisations showed denser clustering of harms among the trenbolone group compared with the non‐trenbolone group.

**Discussion and Conclusions:**

Trenbolone use is associated with a higher prevalence and co‐occurrence of psychosocial and physical concerns relative to other AAS use. These harms suggest trenbolone use is reflective of severe risk profiles within the AAS‐using communities. Targeted harm‐reduction messaging and clinical screening strategies may be warranted for this subgroup.

## Key Point

- Men who use trenbolone reported markedly higher emotional volatility, depressive symptoms, irritability and interpersonal strain than those who use other anabolic‐androgenic steroids.
- Among men who use trenbolone, physical harms, particularly cardiovascular and hepatic concerns, were more prevalent and co‐occurred.
- Data visualisation demonstrated greater clustering of all harms in men who use trenbolone, indicating a more complex health burden.
- Trenbolone use warrants a targeted public health response which includes harm reduction messaging.

## 1 Introduction

Anabolic‐androgenic steroids (AAS) are increasingly used outside medical supervision [1, 2], with non‐medical use increasingly documented across diverse international settings [3, 4]. Prevalence of non‐medical AAS use is higher among men (6.4%) than women (4%) [3, 4]. AAS exposure is associated with a diverse range of psychological and physical harms, including mood instability, aggression and depressive symptoms [5, 6, 7], as well as cardiometabolic strain, hepatic dysfunction and reproductive/endocrine disturbances [8, 9, 10]. Increasingly, attention has turned to whether specific compounds shape the expression and severity of these harms [6, 11, 12]. In particular, trenbolone is frequently described as provoking intense psychological side effects, such as psychological distress and aggression [6, 12]. However, despite accumulating preliminary reports, there remains a shortage of large‐scale, quantitative comparisons that situate trenbolone within the broader AAS landscape across both psychosocial and physical domains. Accordingly, this study compares individuals who use trenbolone alongside other AAS, with those who use AAS but no trenbolone, quantifying the prevalence and co‐occurrence of self‐reported psychosocial and physical concerns.

Trenbolone represents one of the most distinctive and potent compounds in the 19‐Nor AAS class. It is a synthetic AAS with high androgen‐receptor affinity, non‐aromatising properties and notable progestogenic activity [13, 14]. Originally developed for veterinary growth promotion in cattle [15], it later entered human enhancement markets through illicit channels [14]. Trenbolone use is consistently linked with pronounced psychological effects, including heightened irritability and aggression, rapid mood lability, anxiety, dysphoria, sleep disturbance and reduced interest or pleasure in previously enjoyable activities [6, 11, 12, 16]. Emerging preclinical studies have also begun exploring trenbolone's potential neurobiological effects, although these findings derive from animal models and their relevance to humans remains uncertain [17, 18, 19]. It is also associated with a broader physical burden, including cardiometabolic strain, elevations in hepatic enzymes, dermatologic changes, and sexual or reproductive disturbances [14, 16, 20, 21]. Market factors, such as availability, online promotion and cultural positioning as a ‘hard‐edged’ compound, may further concentrate risk [11, 16, 22]. However, much of the trenbolone literature remains restricted to small samples or qualitative reports, leaving critical gaps regarding large‐scale quantitative comparisons within AAS‐using populations and the degree to which harms cluster across psychosocial and physical domains.

These gaps motivate the present study. Although prior quantitative work has identified associations between trenbolone use and specific psychosocial outcomes (e.g., verbal aggression), existing studies have focused on narrower outcome domains and smaller samples. It remains unclear whether trenbolone use is associated with a broader constellation of psychosocial and physical concerns, nor the extent to which these concerns co‐occur within individuals. The present study addresses this gap by examining multi‐domain harm profiles within a large international sample of men who have used AAS in the past 12 months. Leveraging data from the Global Drug Survey 2024 (GDS2024), we compare men who report trenbolone use (trenbolone group) with men who report AAS use without trenbolone (non‐trenbolone group), examining group differences in individual concerns and visualising patterns of co‐occurrence through multiple‐response frequencies and UpSet plots. We anticipated that the trenbolone group would exhibit heavier‐use markers and a higher prevalence of both psychosocial and physical concerns, including greater overlap across domains. We aim to provide a clear, large‐scale delineation of trenbolone's harm profile within an international sample of men who use AAS to inform clinical care and targeted harm‐reduction responses.

## 2 Method

### 2.1 Sample and Procedure

This study employed a cross‐sectional design using self‐report data from the GDS2024, a large‐scale, anonymous online survey conducted annually to examine global patterns of substance use. Recruitment occurred through multiple channels, including mainstream news outlets (e.g., *The Sydney Morning Herald*), social media platforms (e.g., X [formerly Twitter], YouTube) and collaborations with content creators and influencers (e.g., Vigorous Steve) who actively promoted survey participation. The instrument collects extensive sociodemographic data alongside detailed accounts of participants' substance use histories and related outcomes.

The GDS2024 was open for participation between 10 January and 30 April 2024. Completion time varied from approximately 15 to 60 min depending on each participant's substance involvement. Comprehensive methodological information regarding survey design, recruitment and psychometric validation is available in prior publications [23, 24]. Individuals were eligible to participate if they were aged 16 years or older and had used at least one psychoactive drug within the previous 12 months. Participation was entirely anonymous, no names, IP addresses, dates of birth or other identifying details were collected. Reporting follows the STROBE guidelines for observational research.

### 2.2 Measures

The present study utilised demographic information pertaining to age, gender, country of residence, highest education and occupation. Furthermore, questions were asked about participants' AAS usage, including whether they have used AAS within the previous 12 months, their age when first using injectable AAS, oral AAS use (lifetime and prior 12 months), injectable AAS use (lifetime and prior 12 months) and injectable trenbolone use (lifetime and prior 12 months).

The study also gathered data relating to the self‐reported psychosocial and physical concerns associated with AAS use. Example items include ‘physical concern: Hair loss’, and ‘physical concern: Negative impact on heart’; and participants were required to select ‘yes’ or ‘no’ for each. Specifically, the *psychological concerns* section included items pertaining to the following concerns: anger/aggression, depression/low mood, rapid fluctuation in mood and restlessness/irritability. The *physical concerns* section included items assessing concerns pertaining to decreased sexual function, hair loss, negative impacts on sexual organs, decreased fertility, skin condition (e.g., acne) and negative impact on heart.

### 2.3 Analysis

All analyses were conducted using SPSS Version 30 (IBM, 2020), and all graphics were created using Python Version 3.12 (van Rossum, 2024). The primary analysis examined differences in psychosocial concerns and physical concerns between the trenbolone group and non‐trenbolone group. Chi‐squared tests of independence were conducted to determine whether the prevalence of individual psychosocial concerns and physical concerns differed between the group categories. Phi (*ϕ*) coefficients were reported as effect sizes, with *ϕ* = 0.10, 0.30 and 0.50 interpreted as small, medium and large effects, respectively. To examine the distribution and overlap of concerns, multiple response frequency tables were generated for psychosocial concerns and physical concerns, displaying the proportion of responses and cases reporting *yes* for each. Participants could select multiple concerns perceived as resulting from their AAS consumption across psychosocial (8 items; 255 possible response combinations) and physical (12 items; 4095 combinations). Furthermore, the total number of concerns reported per participant was computed, yielding the psychosocial concern pattern (the number of psychosocial concerns each participant endorsed [0 to 8]) and physical concern pattern (the number of physical concerns each participant endorsed [0 to 11]).

The concern variables were binary, whereby a score of 0 or 1 indicated the participant did not or did indicate (respectively) that particular concern. The tables illustrate the extent to which participants experience zero, isolated, or multiple psychosocial and physical side effects. Additionally, UpSet plots were generated to visualise the co‐occurrence of psychosocial concerns and physical concerns across groups. These plots illustrate the intersections and sizes of multiple response sets, providing insight into the co‐occurrence (and frequency) of symptoms in each group. Separate UpSet plots were produced for the trenbolone and non‐trenbolone groups across psychosocial concerns and physical concerns. For all analytical tests, a *p* value of < 0.05 was considered statistically significant. Assumptions for chi‐squared tests were met, as all expected cell frequencies were > 5 in at least 80% of cases.

## 3 Results

### 3.1 Descriptive Statistics

#### 3.1.1 Demographic Characteristics

Data were drawn from 12,615 participants from 117 countries (2615 participants did not provide country data) who responded to the GDS2024, completed the image‐ and performance‐enhancing drug module, and self‐identified as men (*N* = 1146, *M*_age_ = 31.46, SD = 9.93) with a self‐reported history of AAS consumption within the past 12 months. Participants were stratified into two groups, those who self‐reported injectable trenbolone use within the prior 12 months (trenbolone group, *n* = 237), and those who reported no injectable trenbolone use within the prior 12 months (non‐trenbolone group, *n* = 909). Tables 1 and 2 show the frequencies (for categorical demographic characteristics) and descriptive statistics (for continuous demographic characteristics) of the overall sample, respectively.

**TABLE 1:** Frequencies for the categorical AAS usage characteristics of the overall sample (*N* = 1146), the trenbolone group (*n* = 237) and non‐trenbolone group (*n* = 909).

Characteristic	Category	Overall sample *n* (%)	Non‐trenbolone *n* (%)	Trenbolone, *n* (%)	*p*
Lifetime consumption of injectable steroids	Yes	840 (73.3)	605 (66.6)	235 (99.2)	< 0.001
	No	306 (26.7)	304 (33.4)	2 (0.8)	
Consumption of injectable steroids in last 12 months	Yes	774 (67.5)	539 (59.3)	235 (99.2)	< 0.001
	No	372 (32.5)	370 (40.7)	2 (0.8)	
Lifetime consumption of oral steroids	Yes	728 (63.5)	508 (55.9)	220 (92.8)	< 0.001
	No	418 (36.5)	401 (44.1)	17 (7.2)	
Consumption of oral steroids in last 12 months	Yes	538 (46.9)	349 (38.4)	189 (79.7)	< 0.001
	No	608 (53.1)	560 (61.6)	48 (20.3)	

**TABLE 2:** Means and standard deviations for continuous demographic characteristics of the overall sample (*N* = 1146), the trenbolone group (*n* = 237) and non‐trenbolone group (*n* = 909).

Characteristic	*M* (SD)			*p*
	Overall sample	Non‐trenbolone	Trenbolone group	
Age (years)	31.46 (9.93)	31.17 (9.93)	32.57 (9.86)	0.054
Age first used injectable AAS	25.99 (7.61)	26.33 (7.74)	25.20 (7.30)	0.057
Age first used oral AAS	26.45 (7.94)	26.63 (8.11)	26.09 (7.62)	0.413

Abbreviation: AAS, anabolic‐androgenic steroid.

For the categorical demographic variables, there was a significant difference between the groups for lifetime injectable AAS use, *χ* ^*2*^(1) = 102.07, *p* < 0.001, whereby the trenbolone group was far more likely to have ever used injectable AAS (99.2%) compared to the non‐trenbolone group (66.6%). Similarly, a significant difference emerged for injectable AAS use within the last 12 months, *χ* ^*2*^(1) = 136.24, *p* < 0.001, with the trenbolone group again showing higher prevalence (99.2%) than the non‐trenbolone group (59.3%). There was also a significant difference between the groups for lifetime oral steroid use, *χ* ^*2*^(1) = 110.72, *p* < 0.001, whereby the trenbolone group were substantially more likely to have ever used oral AAS (92.8%) relative to the non‐trenbolone group (55.9%). Likewise, oral steroid use within the last 12 months differed significantly between groups, *χ* ^*2*^(1) = 129.07, *p* < 0.001, the trenbolone group reported higher recent oral AAS use (79.7%) compared with the non‐trenbolone group (38.4%). Together, these findings indicate the trenbolone group were more likely to report both oral and injectable AAS use, both historically and within the past year.

No significant differences were observed between groups for chronological age, age at first injectable AAS use, or age at first oral AAS use (all *p* > 0.05; see Table 2 for descriptive statistics).

### 3.2 Psychosocial Concerns

Item‐level frequencies for psychosocial concerns are presented in Table S1. The distribution of the number of psychosocial concerns endorsed per participant (i.e., symptom‐load patterns) is shown in Table S2. Across the full sample, 55.8% of participants did not endorse any psychosocial concerns, 21.5% endorsed one concern and 22.7% endorsed two or more concerns. Among those reporting harms, most endorsed one or two concerns rather than extensive multi‐symptom profiles. The most commonly endorsed concerns were restlessness/irritability (16.2%), depression/low mood (14.4%), rapid mood fluctuation (12.4%) and anger/aggression (11.7%).

#### 3.2.1 Overlap: UpSet Plots

**FIGURE 1:**
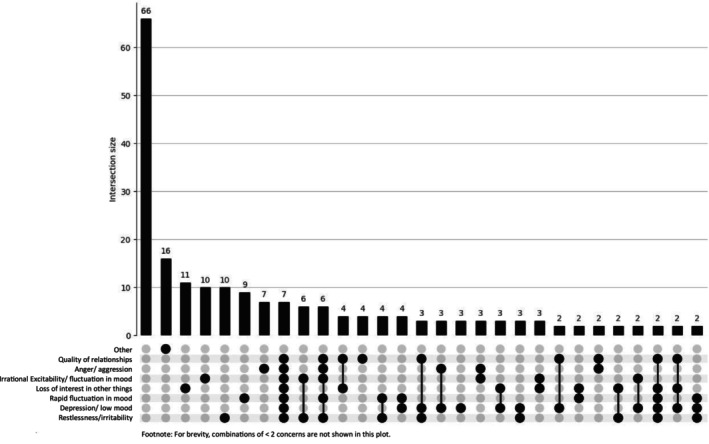
UpSet plot depicting psychosocial concerns for the trenbolone group (*n* = 237). The first bar reflects participants reporting no psychosocial concerns.

**FIGURE 2:**
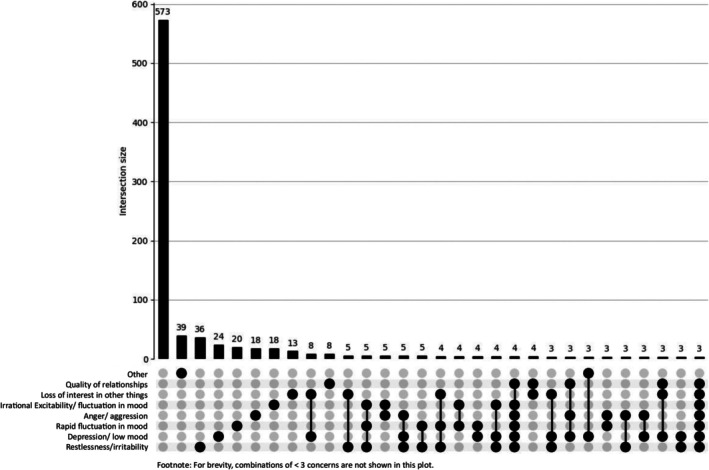
UpSet plot depicting psychosocial concerns for the non‐trenbolone group (*n* = 909). The first bar reflects participants reporting no psychosocial concerns.

As shown in Figures 1 and 2, psychosocial harms did not converge into a single dominant symptom cluster in either group. However, the distribution of higher order intersections was more pronounced among the trenbolone group. The most common multi‐symptom combinations involved irritability, depressive/low mood symptoms, rapid mood fluctuations, anger/aggression and reduced interest in other activities. Although frequency counts were lower due to the smaller sample, the trenbolone group more often endorsed two or more co‐occurring concerns, indicating a more complex affective disturbance profile.

In contrast, the non‐trenbolone group displayed a larger overall volume of psychosocial concerns but a more diffuse pattern, with most co‐occurrence intersections occurring at very low frequencies. These visual patterns align with chi‐squared results showing a significantly higher prevalence of multiple individual psychosocial concerns among the trenbolone group, particularly irritability, fluctuation in mood, depressive affect and interpersonal strain.

### 3.3 Physical Concerns

Item‐level frequencies for physical concerns are presented in Table S3. The distribution of the number of physical concerns endorsed per participant (i.e., symptom‐load patterns) is shown in Table S4. Across the full sample, 36.1% reported no physical concerns, while 63.9% endorsed at least one concern; however, most endorsed one or two concerns rather than extensive multi‐system involvement. The most frequently reported concerns were perceived negative impact on the heart (36.0%), hair loss (30.7%), negative impact on the liver (26.5%) and skin conditions (26.1%).

#### 3.3.1 Overlap: UpSet Plots

**FIGURE 3:**
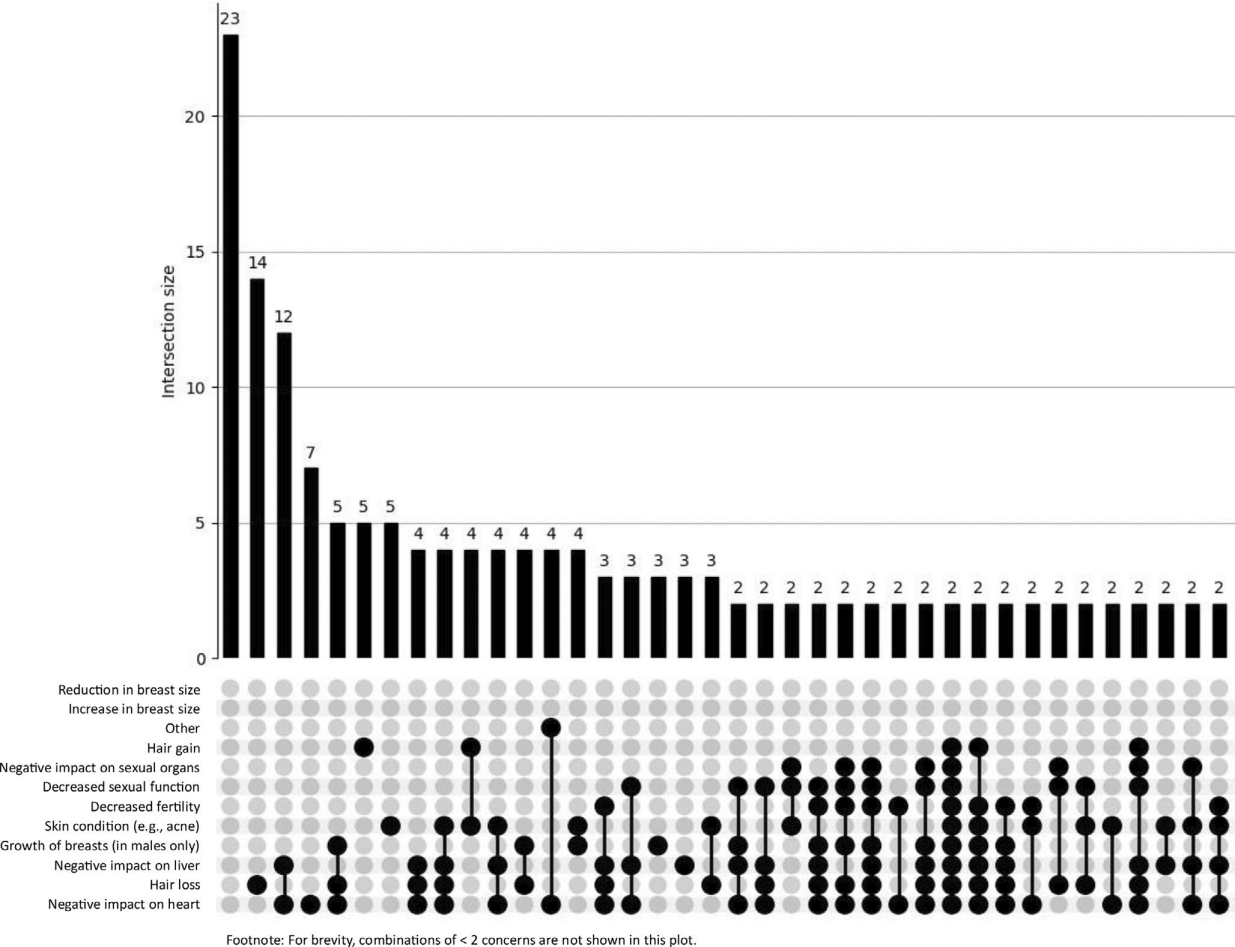
UpSet plot depicting physical concerns for the trenbolone group (*n* = 237). The first bar reflects participants reporting no psychosocial concerns.

**FIGURE 4:**
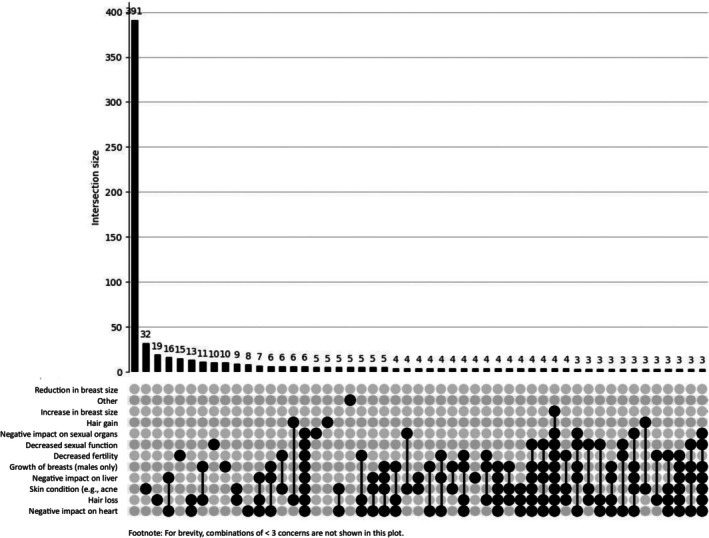
UpSet depicting physical concerns for the non‐trenbolone group (*n* = 909). The first bar reflects participants reporting no psychosocial concerns.

UpSet plots as shown in Figures 3 and 4, physical harms did not cluster into a single dominant pattern in either group. However, high‐order intersections were more pronounced among the trenbolone group even after suppressing low‐frequency combinations (< 2 concerns). The most prevalent multi‐concern intersections in the trenbolone group included combinations involving negative impact on the heart, liver and sexual function, indicating a more concentrated cardiometabolic and endocrine burden.

In contrast, despite a larger overall base rate of physical concerns in the non‐trenbolone group, co‐occurrence patterns were more dispersed, with most intersections occurring at very low frequency (< 10 cases after suppressing < 3 combinations). Together, these findings suggest that while physical harms are widely distributed among men who use AAS generally, trenbolone use is associated with greater clustering of multiple physiological concerns within the same individuals.

### 3.4 Group Comparisons

#### 3.4.1 Psychosocial Concerns

Chi‐squared analyses indicated that the trenbolone group was significantly more likely than the non‐trenbolone group to endorse anger/aggression (*χ* ^*2*^[1] = 25.59, *p* < 0.001, *ϕ* = 0.15), depression/low mood (*χ* ^*2*^[1] = 20.66, *p* < 0.001, *ϕ* = 0.13), rapid mood fluctuations (*χ* ^*2*^[1] = 34.76, *p* < 0.001, *ϕ* = 0.17), irrational excitability/elevation of mood (*χ* ^*2*^[1] = 34.48, *p* < 0.001, *ϕ* = 0.17), restlessness/irritability (*χ* ^*2*^[1] = 36.48, *p* < 0.001, *ϕ* = 0.18), loss of interest in other things (*χ* ^*2*^[1] = 43.64, *p* < 0.001, *ϕ* = 0.20) and relationship difficulties (*χ* ^*2*^[1] = 34.84, *p* < 0.001, *ϕ* = 0.17). All effects were small to approaching‐moderate in magnitude, with the largest differences emerging for loss of interest, irritability and mood instability. No significant group difference was observed for other unspecified psychosocial concerns (*χ* ^*2*^[1] = 2.81, *p* = 0.094, *ϕ* = 0.05). See Table 3 for full group proportions and test statistics.

**TABLE 3:** Group comparisons of psychosocial concerns for the overall sample (*N* = 1146), including the non‐trenbolone group (*n* = 909) and trenbolone group (*n* = 237).

Psychosocial concern	Non‐trenbolone, *n* (%)	Trenbolone, *n* (%)	*χ* ^*2*^ (df = 1)	*p*	ϕ
Anger/aggression	84 (9.2%)	50 (21.1%)	25.59	< 0.001	0.15
Depression/low mood	109 (12.0%)	56 (23.6%)	20.66	< 0.001	0.13
Rapid fluctuation in mood	86 (9.5%)	56 (23.6%)	34.76	< 0.001	0.17
Irrational excitability/elevation of mood	79 (8.7%)	53 (22.4%)	34.48	< 0.001	0.17
Restlessness/irritability	117 (12.9%)	69 (29.1%)	36.48	< 0.001	0.18
Loss of interest in other things	73 (8.0%)	55 (23.2%)	43.64	< 0.001	0.2
Relationship difficulties	69 (7.6%)	49 (20.7%)	34.84	< 0.001	0.17
Other	47 (5.2%)	19 (8.0%)	2.81	0.094	0.05

*Note:* Values represent number (percentage) of participants within each group endorsing the concern. Pearson *χ* ^2^ tests (df = 1) were used to examine between‐group differences. Effect sizes are reported as ϕ.

Collectively, these results indicate that the trenbolone group experienced a markedly broader and more frequent pattern of psychosocial disturbances than the non‐trenbolone group, suggesting a substantially elevated affective burden characterised by emotional volatility, depressive affect and interpersonal strain.

### 3.5 Physical Concerns

Chi‐squared analyses indicated that the trenbolone group was significantly more likely than the non‐trenbolone group to report a wide range of physical concerns, including negative impact on the heart (*χ* ^*2*^[1] = 43.85, *p* < 0.001, *ϕ* = 0.20) and negative impact on the liver (*χ* ^*2*^[1] = 37.63, *p* < 0.001, *ϕ* = 0.18), which showed the largest effects. Significant differences also emerged for hair loss (*χ* ^*2*^[1] = 19.88, *p* < 0.001, *ϕ* = 0.13), gynecomastia (growth of breasts in men; *χ* ^*2*^[1] = 15.06, *p* < 0.001, *ϕ* = 0.12), decreased sexual function (*χ* ^*2*^[1] = 14.22, *p* < 0.001, *ϕ* = 0.11), hair gain (*χ* ^*2*^[1] = 9.55, *p* = 0.002, *ϕ* = 0.09), skin conditions (e.g., acne) (*χ* ^*2*^[1] = 6.34, *p* = 0.012, *ϕ* = 0.07), decreased fertility (*χ* ^*2*^[1] = 5.86, *p* = 0.015, *ϕ* = 0.07), negative impacts on sexual organs (*χ* ^*2*^[1] = 4.66, *p* = 0.031, *ϕ* = 0.06) and other physical concerns (*χ* ^*2*^[1] = 6.32, *p* = 0.012, *ϕ* = 0.07). Effects were small to approaching‐moderate in magnitude overall, with the cardiovascular and hepatic items showing the strongest group separations. No significant differences were observed for reduction in breast size (*χ* ^*2*^[1] = 0.001, *p* = 0.970, *ϕ* < 0.01) or increase in breast size (*χ* ^*2*^[1] = 0.001, *p* = 0.979, *ϕ* < 0.01). See Table 4 for full group proportions and test statistics.

**TABLE 4:** Group comparisons of physical concerns for the overall sample (*N* = 1146), including the non‐trenbolone group (*n* = 909) and trenbolone group (*n* = 237).

Physical concern	Non‐trenbolone, *n* (%)	Trenbolone, *n* (%)	*χ* ^2^ (df = 1)	*p*	ϕ
Decreased sexual function	136 (15.0%)	60 (25.3%)	14.22	< 0.001	0.11
Hair loss	251 (27.6%)	101 (42.6%)	19.88	< 0.001	0.13
Hair gain	77 (8.5%)	36 (15.2%)	9.55	0.002	0.09
Negative impact on sexual organs	109 (12.0%)	41 (17.3%)	4.66	0.031	0.06
Decreased fertility	173 (19.0%)	62 (26.2%)	5.86	0.015	0.07
Gynecomastia	187 (20.6%)	77 (32.5%)	15.06	< 0.001	0.12
Reduction in breast size	4 (0.4%)	1 (0.4%)	0.001	0.97	< 0.01
Increase in breast size	31 (3.4%)	8 (3.4%)	0.001	0.979	< 0.01
Skin condition (acne)	222 (24.4%)	77 (32.5%)	6.34	0.012	0.07
Negative impact on heart	284 (31.2%)	129 (54.4%)	43.85	< 0.001	0.2
Negative impact on liver	204 (22.4%)	100 (42.2%)	37.63	< 0.001	0.18
Other	29 (3.2%)	16 (6.8%)	6.32	0.012	0.07

*Note:* Values represent number (percentage) of participants within each group endorsing the concern. Pearson *χ* ^2^ tests (df = 1) were used to examine between‐group differences. Effect sizes are reported as ϕ. Fisher's Exact Test was consulted where expected cell counts were low.

Taken together, these results indicate the trenbolone group exhibited a broader and more intense physical side‐effect profile, particularly concentrated in cardiometabolic (heart) and hepatic (liver) domains, alongside elevated dermatologic, reproductive and endocrine‐related concerns.

## 4 Discussion

This study examined whether men who use trenbolone alongside other AAS (trenbolone group) report a distinct pattern of harms relative to men who use AAS without trenbolone (non‐trenbolone group). Consistent with our hypotheses, the trenbolone group reported a broader and more frequent pattern of endorsed psychosocial and physical concerns, as well as more pronounced clustering of these concerns within the same individuals. This pattern suggests that trenbolone may exacerbate or compound established AAS‐related risks, placing people who use it at heightened vulnerability to multi‐domain health consequences. Together, these findings offer preliminary but robust quantitative evidence that trenbolone use is associated with a concentrated multi‐symptom profile, extending prior clinic‐based and qualitative observations into a large, international community sample.

Beyond group differences, the broader AAS‐using sample displayed a heterogeneous but generally moderate symptom profile. Over half of participants did not endorse any psychosocial concerns, and more than one‐third reported no physical concerns. Among those reporting harms, most endorsed one or two concerns rather than extensive multi‐system involvement. Thus, while trenbolone use appears to shift risk upward and concentrate the likelihood of reporting multiple concerns, adverse outcomes were not universal among people who use AAS. This pattern indicates that although non‐medical AAS use is associated with a range of self‐reported adverse effects, highly clustered or pervasive symptom profiles were less common at the overall sample level within this enhancement‐focused population.

Psychosocial concerns were more commonly reported in the trenbolone group across all emotional and interpersonal domains assessed. Group differences were strongest for irritability, rapid mood instability, depressive affect and reduced interest in other activities, patterns that align closely with androgen‐sensitive neural systems underpinning arousal, affect regulation and reward processing [6, 7, 25]. Trenbolone's high androgen‐receptor affinity and progestogenic activity may amplify these disruptions, potentially heightening vulnerability to interpersonal conflict and functional impairment [13, 14]. Furthermore, these findings align with and extend existing literature describing heightened psychosocial burden associated with trenbolone use, including psychological distress, aggression and cognitive disturbance [6, 12, 21]. Visual patterns from the UpSet plots reinforce this interpretation. Although concerns did not consolidate into a single dominant cluster, the trenbolone group more frequently endorsed multiple co‐occurring concerns, indicating a more complex affective disturbance profile. Taken together, these findings suggest men who use trenbolone experience disproportionately severe psychological consequences that extend beyond the expected effects of non‐medical AAS use alone.

Similarly, the trenbolone group reported substantially higher rates of physical concerns spanning cardiometabolic, hepatic, dermatologic and reproductive systems. The largest group differences emerged for perceived negative impacts on the heart and liver, which are patterns consistent with trenbolone's high androgen‐receptor affinity and metabolic potency [14, 20, 26]. This pattern is also consistent with emerging clinic‐based observations that trenbolone consumption may exacerbate cardiovascular strain relative to other AAS, particularly among individuals engaging in high‐dose stacking practices [14, 20, 21]. Furthermore, this broadly aligns with mechanistic evidence indicating that trenbolone can influence hepatic metabolism by upregulating enzymes involved in mitochondrial fatty‐acid oxidation, including enoyl‐CoA hydratase and acyl‐CoA dehydrogenase [14, 27]. As such, trenbolone may heighten hepatocellular stress and downstream endocrine disruption when used in stacked or high‐dose regimens [20, 21]. The trenbolone group's substantially greater oral AAS involvement likely compounds this effect, given oral formulations are more directly hepatotoxic and increase hepatic metabolic load [28]. UpSet plot visualisations reinforce this interpretation, showing that cardiometabolic, hepatic and reproductive concerns co‐occurred more densely within the trenbolone group. Collectively, these patterns suggest trenbolone may intensify the systemic consequences of non‐medical AAS use, placing men who use trenbolone at increased risk of long‐term morbidity, particularly within cardiovascular and hepatic domains.

### 4.1 Implications

From a harm‐reduction perspective, these findings highlight the importance of compound‐specific awareness. While many AAS are associated with adverse psychological and physiological effects, preclinical and mechanistic studies suggest trenbolone may exert disproportionately severe neurotoxic and systemic impacts relative to several other AAS, including testosterone and certain dihydrotestosterone derivatives. Experimental evidence suggests that trenbolone may exert disproportionate neurotoxic effects relative to testosterone or dihydrotestosterone derivatives, including blood–brain barrier penetration and pro‐apoptotic activity in neuronal tissue [14, 29], as well as structural disruption in animal models [30]. These findings provide biological plausibility for the heightened psychosocial risk profile observed here.

Accordingly, men who use trenbolone may represent a higher risk subgroup within the broader AAS‐using population. Although AAS use is not routinely screened in many healthcare settings, these findings underscore the potential value of increased clinician awareness of compound‐specific risk patterns. Incorporating compound‐level enquiry, particularly regarding trenbolone, may support earlier identification of emerging psychiatric or cardiometabolic complications and facilitate more targeted harm‐reduction strategies.

### 4.2 Strengths and Limitations

A strength of this work is the large, international sample, providing sufficient power to characterise men who use trenbolone as a distinct subgroup. Unlike studies focused on narrow clinical cohorts, these data capture real‐world enhancement practices, including heavier and more complex patterns that rarely present to healthcare. Our co‐occurrence approach moves beyond single‐outcome reporting to model overlapping harms, offering a more ecologically valid estimate of total symptom load. Use of established GDS analytic frameworks further enhances comparability across years and platforms.

However, interpretation is limited by self‐reported, cross‐sectional data, which cannot establish causality and may be influenced by recall or symptom‐awareness differences. Outcomes represent perceived effects rather than clinically verified diagnoses, and we were unable to assess severity, onset or persistence. In addition, the survey assessed a predefined list of psychosocial and physical concerns; therefore, participants classified as reporting no harms were those who did not endorse any of the listed items. It remains possible that some individuals experienced adverse effects not captured by the survey measures or that certain harms may not have resonated with all participants. We also did not incorporate available dosing variables, such as weekly milligram ranges, cycle length, stacking patterns or prescription status, which likely moderate risk and warrant dedicated modelling. Recruitment strategies may over‐represent digitally connected consumers and under‐sample those more marginalised from services. Furthermore, as GDS is a self‐selecting survey, people who use illicit psychoactive substances may be over‐represented relative to the general population. However, previous methodological work suggests that such samples remain valuable for examining patterns of drug use and associated health outcomes within specific populations when analyses focus on internal comparisons rather than prevalence estimation [23]. In the present study, analyses compared men who had used trenbolone within the past 12 months, and those who had not, within the same sampling frame of men reporting past‐year AAS use, meaning internal group contrasts remain interpretable despite limits to broader population generalisability. The study also draws on one of the largest international samples of men who use AAS collected to date.

## 5 Conclusion

This study provides the first large‐scale international evidence that trenbolone use is associated with a more frequent, broader and more tightly clustered constellation of psychosocial and physical concerns than using other AAS alone. These findings extend prior clinical observations by demonstrating trenbolone's distinct harm profile within an international enhancement‐focused population. Future research using biomarker verification, detailed dosing and administration data, and longitudinal follow‐up is needed to clarify escalation pathways and recovery trajectories. Given trenbolone's strong cultural foothold within online bodybuilding and performance‐enhancement communities, targeted prevention strategies and clinical response frameworks for this high‐risk subgroup should be prioritised to mitigate downstream morbidity.

## Author Contributions

Each author certifies that their contribution to this work meets the standards of the International Committee of Medical Journal Editors. B.B. led the formal analysis, data curation, visualisation, preparation of the original draft and contributed to review, editing and project administration. B.A., C.P., A.B., K.T.G. and J.M.N. contributed to the interpretation of findings and critically revised the manuscript for important intellectual content. J.F. provided methodological guidance, statistical supervision, interpretation of findings and manuscript review. A.W. contributed to study design input, data acquisition for the Global Drug Survey, interpretation and manuscript review. A.B. contributed domain expertise relating to steroid markets and consumer practices, as well as manuscript review and interpretation. T.P. contributed to conceptualisation, study design, supervision, interpretation of findings, lived‐living experience expertise, cultural safety and review and editing of the manuscript. All authors approved the final version and agree to be accountable for all aspects of the work.

## Funding

T.P. supported by a National Health and Medical Research Council Investigator Grant (2041822).

## Conflicts of Interest

The authors declare no conflicts of interest.

## Supporting information


**Table S1:** Multiple‐response frequencies for psychosocial concerns across the full sample of men reporting past‐12‐month AAS use (*N* = 1146).
**Table S2:** Psychosocial concerns pattern table displaying the number of participants (*N* = 1146) reporting each psychosocial concern.
**Table S3:** Multiple‐response frequencies for physical concerns across the full sample of men reporting past‐12‐month AAS use (*N* = 1146).
**Table S4:** Physical concerns pattern table displaying the number of participants (*N* = 1146) reporting each psychosocial concern.

## Data Availability

The data that support the findings of this study are available on request from the corresponding author. The data are not publicly available due to privacy or ethical restrictions.
