# Supplementary material for: The Trenbolo(g)ne Sandwich: An International Study Comparing Health Harms Among Men Who Use Anabolic‐Androgenic Steroids With and Without Trenbolone
Source: Drug Alcohol Rev. 2026 Apr 27;45:e70162. doi: 10.1111/dar.70162 (PMC13112052; doi:10.1111/dar.70162)
Supplement: Supplementary file 1 — Table S1: Multiple‐response frequencies for psychosocial concerns across the full sample of men reporting past‐12‐month AAS use (N = 1146). Table S2: Psychosocial concerns pattern table displaying the number of participants (N = 1146) reporting each psychosocial concern. Table S3: Multiple‐response frequencies for physical concerns across the full sample of men reporting past‐12‐month AAS use (N = 1146). Table S4: Physical concerns pattern table displaying the number of participants (N = 1146) reporting each psychosocial concern. [file DAR-45-0-s001.docx]

**Table S1**

*Multiple-response frequencies for psychosocial concerns across the full sample of men reporting past-12-month AAS use (N = 1,146).*

| Psychosocial Concern | Frequency | % of Responses | % of Cases |
| --- | --- | --- | --- |
| Restlessness/irritability | 186 | 17.4 | 16.2 |
| Depression/low mood | 165 | 15.4 | 14.4 |
| Rapid fluctuation in mood | 142 | 13.3 | 12.4 |
| Irrational excitability/elevation of mood | 132 | 12.3 | 11.5 |
| Anger/aggression | 134 | 12.5 | 11.7 |
| Loss of interest in other things | 128 | 11.9 | 11.2 |
| Quality of my relationship with others | 118 | 11 | 10.3 |
| Other | 66 | 6.2 | 5.8 |
| Total | 1071 | 100 |  |

**Note.** Participants could endorse multiple psychosocial concerns. “% of responses” represents the proportion of total endorsements across all listed concerns (denominator = total number of concern selections). “% of cases” represents the proportion of participants (N = 1146) who endorsed each concern.

**Table S2**

*Psychosocial concerns pattern table displaying the number of participants (N = 1146) reporting each psychosocial concern.*

| Number of Psychosocial Concerns | Frequency | Percent | Cumulative Percent |
| --- | --- | --- | --- |
| 0 | 639 | 55.8 | 55.8 |
| 1 | 246 | 21.5 | 77.3 |
| 2 | 114 | 9.9 | 87.2 |
| 3 | 68 | 5.9 | 93.1 |
| 4 | 37 | 3.2 | 96.3 |
| 5 | 19 | 1.7 | 98 |
| 6 | 12 | 1 | 99 |
| 7 | 10 | 0.9 | 99.9 |
| 8 | 1 | 0.1 | 100 |
| Total | 1146 | 100 |  |

**Table S3**

*Multiple-response frequencies for physical concerns across the full sample of men reporting past-12-month AAS use (N = 1,146).*

| Physical Concern | Frequency | % of Responses | % of Cases |
| --- | --- | --- | --- |
| Decreased sexual function | 196 | 8.1 | 17.1 |
| Hair loss | 352 | 14.6 | 30.7 |
| Hair gain | 113 | 4.7 | 9.9 |
| Negative impact(s) on sexual organs | 150 | 6.2 | 13.1 |
| Decreased fertility | 235 | 9.7 | 20.5 |
| Growth of breasts (in males only) | 264 | 10.9 | 23 |
| Reduction in breast size | 5 | 0.2 | 0.4 |
| Increase in breast size | 39 | 1.6 | 3.4 |
| Skin condition (e.g., acne) | 299 | 12.4 | 26.1 |
| Negative impact on heart | 413 | 17.1 | 36 |
| Negative impact on liver | 304 | 12.6 | 26.5 |
| Other | 45 | 1.9 | 3.9 |
| Total | 2415 | 100 |  |

**Note.** Participants could endorse multiple physical concerns. “% of responses” represents the proportion of total endorsements across all listed concerns (denominator = total number of concern selections). “% of cases” represents the proportion of participants (N = 1146) who endorsed each concern.

**Table S4**

*Physical concerns pattern table displaying the number of participants (N = 1146) reporting each psychosocial concern.*

| Number of Physical Concerns | Frequency | Percent | Cumulative Percent |
| --- | --- | --- | --- |
| 0 | 414 | 36.1 | 36.1 |
| 1 | 156 | 13.6 | 49.7 |
| 2 | 162 | 14.1 | 63.8 |
| 3 | 138 | 12 | 75.8 |
| 4 | 86 | 7.5 | 83.3 |
| 5 | 71 | 6.2 | 89.5 |
| 6 | 58 | 5.1 | 94.6 |
| 7 | 30 | 2.6 | 97.2 |
| 8 | 20 | 1.7 | 98.9 |
| 9 | 9 | 0.8 | 99.7 |
| 11 | 1 | 0.1 | 99.8 |
| 12 | 1 | 0.1 | 99.9 |
| Total | 1146 | 100 |  |
